# Is the Devil in *h*?

**DOI:** 10.3390/e23050632

**Published:** 2021-05-19

**Authors:** Andrei Khrennikov

**Affiliations:** International Center for Mathematical Modeling in Physics and Cognitive Sciences, Linnaeus University, SE-351 95 Växjö, Sweden; Andrei.Khrennikov@lnu.se

**Keywords:** complementarity principle, heisenberg uncertainty principle, copenhagen interpretation, quantum nonlocality, bell nonlocality, luders nonlocality, bohr quantum principle, fundamental principles of quantum mechanics, indivisible quantum of action, special relativity, constancy of light’s velocity

## Abstract

This note is a part of my effort to rid quantum mechanics (QM) nonlocality. Quantum nonlocality is a two faced Janus: one face is a genuine quantum mechanical nonlocality (defined by the Lüders’ projection postulate). Another face is the nonlocality of the hidden variables model that was invented by Bell. This paper is devoted the deconstruction of the latter. The main casualty of Bell’s model is that it straightforwardly contradicts Heisenberg’s uncertainty and Bohr’s complementarity principles generally. Thus, we do not criticize the derivation or interpretation of the Bell inequality (as was done by numerous authors). Our critique is directed against the model as such. The original Einstein-Podolsky-Rosen (EPR) argument assumed the Heisenberg’s principle without questioning its validity. Hence, the arguments of EPR and Bell differ crucially, and it is necessary to establish the physical ground of the aforementioned principles. This is the quantum postulate: the existence of an indivisible quantum of action given by the Planck constant. Bell’s approach with hidden variables implicitly implies rejection of the quantum postulate, since the latter is the basis of the reference principles.

## 1. Introduction

Recently I published a series of papers which can be unified by the slogan “getting rid of nonlocality from quantum physics” [1,2,3,4,5] (also see recent papers of References [6,7,8] for a discussion). The aim is to decouple nonlocality from quantum theory. The wide use of the notion of quantum nonlocality overshadows the real result of quantum theory, mystifies it, and generates unjustified expectations and speculative interviews for the mass-media by otherwise very respectable scientists. The main message of aforementioned papers is that quantum theory is local, that “spooky action at a distance” was just Einstein’s catchy slogan [9] from a letter to Born in 1947 [10]. Einstein directed it against *the individual interpretation* of a quantum state. This interpretation is often referred as *the Copenhagen interpretation* of QM. From his viewpoint, one should either reject this interpretation or confront spooky action at a distance: (see Reference [5] for details and a probabilistic analysis). This viewpoint was especially clearly presented in an exchange of letters between Einstein and Schrödinger [11].

One of the complications in ridding nonlocality from QM is that so-called “quantum nonlocality” is a two-faced Janus [5]. People freely refer to his different faces, mix them, and often cannot distinguish them. The two faces of nonlocality of Janus are:

- *Lüders nonlocalty:* apparent nonlocality of QM based on the projection postulate [12] and discussed in the EPR [13]; and
- *Bell nonlocality:* subquantum nonlocality based on a misleading interpretation of violation of the Bell inequalities [14,15,16,17].

Typically, in saying “quantum nonlocality”, one does not specify whether this is a Lüders or Bell nonlocalty (often the author does not even understand the difference between these nonlocalities). So, the first step to elimination of nonlocality from quantum theory is taking into account its Janus-like structure [5].

In fact, the most consistent representation of Lüders nonlocalty nonlocality can be found in Aspect’s papers [18,19]. Aspect did not refer to EPR “elements of reality” and he proceeded straightforwardly with the Lüders projection postulate [12]. We remark that the projection postulate is often referred as the von Neumann-Lüders postulate or even simply the von Neumann postulate. However, von Neumann [20] sharply distinguished between the cases of observables with non-degenerate and degenerate spectra. For the latter case, he considered a more general form of the state transformation process generated by observation retro-action; in particular, according to von Neumann a pure initial state can be transformed into a mixed state, as in the modern theory of quantum instruments [21,22]. EPR used only the projection postulate for arbitrary observables, i.e., as was later formalized by Lüders [12].

In Reference [1], violation of the Bell inequalities was treated in a purely quantum framework, i.e., without coupling to hidden variables (cf. References [9,23,24,25,26,27,28,29]). What does quantum theory say about (non)violation of the Bell inequalities? In this framework, violation versus satisfaction of such inequalities is equivalent to *local incompatibility versus compatibility of observables.* These inequalities should be treated as statistical tests for the complementarity principle (see Section 8).

However, one can say that the essence of these inequalities is in their derivation on the basis of the Bell model with hidden variables [14,15,16,17]. In this paper, we want to terminate this line of thinking by showing that the Bell hidden variables project is in striking contradiction with the foundations of quantum mechanics. To see this, one need not to derive any new inequality. From the very beginning (already by setting the hidden variable model), it is clear that *Bell’s model conflicts with the fundamental principle of QM, the Bohr complementarity principle* (see Bohr [30], as well as References [31,32,33]), and, in particular, the Heisenberg uncertainty principle. Thus, now, we do not criticize the derivations and interpretations of the Bell type inequalities (cf. References [34,35,36,37,38,39,40,41,42]). We stress that, by starting with the Bell hidden variables, one goes on a Crusade against complementarity.

If one accepts this viewpoint, then the following natural question immediately arises: *Why should the complementarity principle be violated only for compound systems?* (If it were violated.) It seems that there is no reason for this. And studies on intrasystem entanglement (of the degrees of freedom of say a single atom) and, in particular, classical entanglement (of the degrees of freedom of say classical electromagnetic field) confirm this viewpoint (see Reference [43] for a review and Reference [4] for coupling of classical entanglement and complementarity).

It is often said that the aim of the Bell hidden variables project was explanation of long distance correlations; we discuss this question in Section 5. We stress that Bell’s attempt of explanation is based on the rejection of the complementarity principle, that the price of such an attempt is too high.

By struggling with Bell nonlocality and opposing it to the complementarity principle, it is worthwhile to find the physical ground of this principle. It is the Bohr *quantum postulate* [30,44,45,46] declaring the existence of an indivisible quantum of action (Planck quantum). Thus, in my view, the resolution of the endless debates on quantum nonlocality, action at a distance, and the Bell inequalities is possible only on the basis of this postulate. This crucial issue is totally missed in these debates. Recently this issue was discussed in Reference [47]. The present paper can be considered as an essential extension of argument from Reference [47] with inclusion of the discussion of a few related foundational problems, as quantum nonlocality, Bell’s inequality, the structure of classical and quantum probability theories, and their recent updates, the projection postulate, EPR-argument, comparison with special relativity, Zeilinger’s search for a fundamental principle of quantum mechanics, and pre-quantum models, such as mine (for the latter, see Appendix B).

We recall (in Section 7) Bohr’s and Heinsenberg’s viewpoints on the quantum postulate, especially Heinsenberg’s comparison of the existence of the quantum of action (h≠0) with finiteness of the speed of light (c<∞) and its independence of the inertial frame [30,44,45].

In Section 7, we recall the practically forgotten paper of Zeilinger [48] in which he looked for the fundamental principles of QM similar to the principles of special relativity. The quantum postulate definitely plays the crucial role in formulation of these fundamental principles (Section 7). This postulate can be considered as the physical core of the Bohr principle of complementarity. The latter is one of the fundamental principles of QM. However, this principle is about observations, the way of extracting of information about quantum systems. This is an epistemological principle [31,32,49,50]. As Bohr himself finally concluded, his quantum postulate is ontic, it is about physical reality as it is.

During his life, Bohr presented several versions of the complementarity principle. In References [3,5,51], I expressed my vision on Bohr’s ideas as the block of sub-principles (see Section 8). I think that such a compact formulation of Bohr’s principles is important for further discussions of the type “Bohr vs. Bell” [3]. Today, the Bohr complementarity principle is discussed mainly by philosophers, e.g., in References [31,32,33]; see, however, e.g., Jaeger et al. [52] for technical studies.)

Similarly to Einstein’s formulation of the relativity principle on the speed of light [53], we formulate the quantum action principle based of the Bohr quantum postulate (Section 7). Following von Weizsäcker [54] and Atmanspacher and Primas [49,50], we consider QM as an epistemic theory, a theory about knowledge (also see References [55,56]). So, the quantum action principle is the epistemic counterpart of Bohr’s quantum postulate of the existence of an indivisible quantum of action, but this postulate has an ontic nature.

This is a good place to recall the recent attempts to derive the quantum formalism from “natural probabilistic and information principles”, e.g., in References [57,58,59,60]. This activity differs from our attempt (following Zeilinger [48]) to formulate the fundamental principles of QM. We do not try to derive its mathematical formalism. The latter can be compared with the dressing for the main dish, we want to taste the dish without dressing.

We start with the comparative analysis of the views of Einstein, Podolsky, and Rosen and Bell (Section 2 and Section 3). Typically, one considers Bell as the follower of EPR and claims that the Bell inequality is straightforwardly related to the EPR-pradox. It seems to me that this viewpoint is misleading. Then, we point (Section 4) that, by considering Bell’s hidden variables model, one struggles against the complementarity principle and, in fact, against the existence of the Planck quantum of action.

Would one like to “explain” long distance correlations at the cost of again confronting the ultraviolet (Rayleigh–Jeans) catastrophe and rejecting Planck’s original work on black body radiation?

## 2. EPR

We start with recollection of the basic notion of the EPR in Reference [13], an element of reality:“If, without in any way disturbing a system, we can predict with certainty (i.e., with probability equal to unity) the value of a physical quantity, then there exists an element of reality corresponding to that quantity”.

Now, we point out to the commonly forgotten historical fact that *the EPR paper was directed against the Copenhagen interpretation of the wave function* [13]. Since this interpretation has many versions (Plotnitsky even proposed to speak about interpretations in the spirit of Copenhagen [31,32]), it is important to specify the EPR treatment of this interpretation.

**Copenhagen interpretation** (EPR) *The wave function (quantum state) ψ represents the state of an individual quantum system.*

It is important to stress that “state” is interpreted epistemically as representing knowledge about possible outcomes of measurements on the system in the state ψ. So, ψ is not an ontic state-not the state of the system as it is, i.e., without relation to external observations. The state interpretation in the EPR-paper is very close to the modern information interpretations used in quantum information theory. This point has not been so much highlighted (see, however, References [49,50,61,62]).

By this interpretation, the quantum mechanical description based on the wave function representation of the state of a quantum system is complete. The complete physical theory is defined as follows [13]: *any element of physical reality has a counterpart in the physical theory.*

The EPR-reasoning was, thus, based on two basic quantum mechanical principles:

- reduction of the wave function (the projection postulate) resulting from measurement retro-action; and
- the Heisenberg uncertainty principle.

The latter was formulated as follows: *“It is shown in quantum mechanics that, if the operators corresponding to two physical quantities, say A and B, do not commute, AB≠BA, then the precise knowledge of one of them precludes such knowledge of the other. Furthermore, any attempt to determine the latter experimentally will alter the state of the system in such a way as to destroy the knowledge of the first.”*

EPR showed that the assumption that QM (following the Copenhagen interpretation) is a complete theory implies violation of the Heisenberg uncertainty principle. Since they were sure of the validity of this principle, EPR concluded that the quantum mechanical description of nature is incomplete.

EPR did not question the validity of the Heisenberg principle. If it would be possible to violate this principle, then assigning to the same system two wave functions which are eigenfunctions of observables represented by non-commutative operators would not lead to any problem.

Thus, by concluding that *“…the wave function does not provide a complete description of the physical reality, we left open the question of whether or not such a description exists” and believing “…that such a theory is possible”,* they do not dream of a theory violating the Heisenberg’s uncertainty principle. By reading later works of Einstein, we can guess that he wanted to construct a classical field model underlying QM [63] (see Appendix B for such an attempt). *EPR also pointed out that nonlocality is an alternative to this conclusion, an alternative that they reject.*

The proponents of EPR did not question the validity of the quantum mechanical description, they were just looking for a more detailed description. But, this deeper description should respect the basic principles of QM, including the uncertainty and complementarity principles.

Now, we move to the Bohr’s reply [64] to the EPR [13]. In this reply, Bohr endowed the Heisenberg uncertainty principle with the interpretation based on the complementarity principle. In this interpretation, Bohr highlighted the contextuality counterpart of this principle (see Section 8, the principle of contextuality). He pointed out that EPR argumentation is based on consideration of two incompatible experimental arrangements (contexts) for measurements on the compound system $S=(S_{1},S_{2}):$

- **Position measurement.** The measurement of the position of $S_{2}$ through the measurement the position of $S_{1}.$
- **Momentum measurement.** The measurement of the momentum of $S_{2}$ through the measurement the momentum of $S_{1}.$

This contextual counterargument against the EPR-argument was preceded by the analysis of diffraction of a noncompound system. By this analysis, Bohr showed that for a single particle its position (slit) and momentum (point at the registration screen) correspond to two different experimental contexts, so they cannot be treated as properties of a particle. Then, by moving to the EPR situation he pointed out that there is no difference between measurements of incompatible observables of a single particle or a compound system. Roughly speaking, Bohr wanted to say that if even for a single particle position and momentum are irreducibly coupled to corresponding experimental contexts, then there is no reason to expect something cardinally different for a compound system of two particles. This line of thinking is important for foundational analysis of the Bell-argument and experiments based on violation of the Bell type inequalities (see References [1,2,3,4,5]).

## 3. Bell

Although Bell started his paper, Reference [14], by referring to the EPR paper as proving the incompleteness of QM, his model with hidden variables has not so much to do with the EPR “dream” of a complete physical theory generalizing QM. It is surprising that this inconsistency has never been emphasized in numerous papers on Bell’s inequality (see, e.g., Aspect [18,19]). The main difference of Bell’s model from the EPR-dream is that his model is in the striking contradiction with the quantum mechanical description, especially with the Heisenberg uncertainty principle.

Consider Bell’s random variables A(a,λ),B(b,λ) representing observables of Alice and Bob, respectively. Surprisingly, Bell did not highlighted that, besides probabilities

(1) $$p_{a,b}\left(x,y\right)=p\left(A\left(a,\lambda \right)=x,B\left(b,\lambda \right)=y\right)$$

for compatible observables, Bell’s model describes probabilities

(2) $$p_{a,a^{\prime }}\left(x_{1},x_{2}\right)=p\left(A\left(a,\lambda \right)=x_{1},A\left(a^{\prime },\lambda \right)=x_{2}\right)$$

for generally incompatible observables represented by noncommuting operators. (This problem is especially clear in consideration of the inequality of Clauser et al. (CHSH) [17].) From the very beginning, i.e., without any Bell type inequality, this assumption contradicts to the QM-representation of observables and, hence, to the Heisenberg uncertainty principle (or, more generally to the Bohr complementarity principle).

Of course, one may proceed with subquantum models, but *without identification of the values of random variables with values of quantum observables and without identification of “hidden correlations” with the experimental correlations.* And De Broglie emphasized [65] this viewpoint. However, people wanted experimental verification…

Thus, from the very beginning Bell’s model of hidden variables was designed as contradicting the uncertainty principle. Therefore, it is not surprising that, as was shown in my recent paper, Reference [1], violation-satisfaction of the CHSH-inequality can be formulated in terms of noncommutativity-commutativity of operators representing local observables of Alice and Bob, respectively, as indicated above.

## 4. Against Complementarity?

The Heisenberg uncertainty principle was the starting point for Bohr’s formulation of the complementarity principle [44,45,46] (see my recent papers, References [1,3,51], a “gentle” presentation of this principle for non-philosophers; also see Section 8). Thus, in the light of the above consideration, we can say that, in fact, Bell’s argument was directed against the Bohr complementarity principle. This argumenty was overshadowed by the nonlocality issue (which is irrelevant to the problem, at least from the viewpoint of EPR and the author of this paper). Of course, rejection of the complementarity principle (or the Heisenberg uncertainty principle) would have similar catastrophic consequences even for non-compound systems (see Bohr’s reply [64] to EPR and the discussion at the very end of Section 2), say a single atom or neutron, as we can see from the contextuality tests (see, e.g., Reference [66]).

To discard the Bell model with hidden variables, one need not to derive inequalities and test them experimentally. Of course, one should believe in the basic principles of QM. (In the opposite case, she should say explicitly about this, about her battle against the quantum postulate). The main impact of experimental tests [61,67,68,69,70] is a demonstration that quantum correlations (predicted by QM) are preserved over long distances. The latter plays the crucial role in quantum engineering. However, correlation preservation can be checked directly without inequalities. Moreover, by operating with the CHSH-combination of correlations experimenter can miss mutual compensation of deviations from QM. In Aspect’s pioneer experiment [71], the correlations did not match the quantum prediction, but they mystically compensated each other to violate the Bell inequality (see Reference [72] for discussion). (In spite of numerous discussions with experimenters, I am still not sure that data from the basic experiments on say CHSH-inequality is clean from the mentioned Aspect-type anomaly. Papers typically present only the CHSH-correlation combination but not separate correlations for pairs of experimental settings).

## 5. Explaining: Long Distance Correlations vs. Violation of the Complementarity Principle

Typically, followers of the Bell argument (that has not so much to do with the original EPR-argument) want to explain long distance correlations. I think that the essence of the problem is in the word “explain”.

In science, we operate with mathematical models of physical processes. So, “explain” means “to describe by some mathematical model”. And quantum mathematics, as a mathematical model, describes perfectly well the long distance correlations: entangled states and projection type measurements. So, it seems that Bell and his followers had something different in mind.

*Why was Bell not satisfied with the quantum mechanical description?*

From reading Bell, I have the impression that he “simply” wanted to re-establish the realism of classical physics. But, what is the main quantum barrier for such realism? Everybody knows this very well, this is the Bohr complementarity principle with starting point from the Heisenberg uncertainty relation; see Bohr [44], *“…an independent reality in the ordinary physical sense can neither be ascribed to the phenomena nor to the agencies of observation.”* This is clearly stated in the EPR Reference [13]. Bell and his followers should have said something like the following: *we want to disprove the Heisenberg uncertainty relations.* Unfortunately, it was never stated explicitly. Instead, people operate with such an ambiguous notion as “local realism”.

Suppose somebody, say Alice, questions the Heisenberg uncertainty principle. Then, why should she consider compound systems? Does she think that this principle is violated only for compound systems? This would be really strange. Thus, before trying to explain the long distance correlations with the Bell-type hidden variables model, it would be reasonable to try explain the incompatibility of observables and their corresponding spin projections to different axes or incompatibility of position and momentum observables.

The main feature of the Bell model with hidden variables, the feature crying for justification, is violation of the complementarity principle. It is not so natural to try to“explain” long distance correlations without any attempt to explain violation of this principle.

## 6. The Root of Complementarity: The Devil Is in the Planck Constant

Thus, by starting the anti-complementarity battle it is useful to remind the foundational roots of complementarity. The Bohr’s complementarity principle will be discussed in detail in Section 8.

For Bohr, the root of the complementarity is the existence of *indivisible quantum of action* given by the Planck constant h. The existence of this quantum prevents the total separation of the genuine physical features of a system from the properties of interaction with a measurement apparatus. So, the seed of the Bohr complementarity principle is the Planck constant h.

It is meaningless to start a Crusade against complementarity without trying to understand the origin of this fundamental quantum of action in nature. Neither Einstein nor Bell tried to perform such investigation; in fact, neither Bohr nor Heisenberg, for them this is just the feature of nature, such as, e.g., the constancy of the speed of light. And, for the moment, this position can be considered as the only one possible.

## 7. Quantum Action Principle

We recall that Zeilinger was looking for the fundamental principle of QM [48], similar to Einstein’s principle of relativity:

*The laws of physics are invariant (i.e., identical) in all inertial frames of reference.*

And he formulated the following principle of the quantization of information:

*An elementary system represents the truth value of one proposition.*

Surprisingly, in his paper, Reference [48], Zeilinger did not mention the Bohr complementarity principle. In fact, Zeilinger’s postulate is nothing else than Bohr’s statement about the quantum phenomenon. The latter can be considered as a part of the complementarity principle (see, especially, my recent paper, Reference [4]). We shall be back to this issue in Section 8.

Now, we recall that the theory of special relativity is based on two Einstein’s principles, of which the second one is about light’s velocity:

*The speed of light in a vacuum is the same for all observers, regardless of the motion of the light source or observer.*

(In particular, this principle presumes finiteness of light’s velocity.) We now point to the close quantum analog of this principle.

Bohr stressed [44] that the essence of quantum theory *“may be expressed in the so-called quantum postulate, which attributes to any atomic process an essential discontinuity, or rather individuality, completely foreign to the classical theories and symbolised by Planck’s quantum of action.”* On the basis of Bohr’s quantum postulate, we formulate the following principle of QM that can be considered as the analog of Einstein’s second principle:

**Quantum action principle:** *The quantum of action is the same for all observers, regardless experimental contexts.*

We can say that this principle is the epistemic counterpart of the Bohr’s quantum postulate. The formulation of the quantum action principle involves observables, but the quantum postulate, the existence in nature of indivisible quantum of action, is about nature as it is, i.e., this is the ontic postulate.

Today, it is practically forgotten that, by formulating the uncertainty principle, Heisenberg pointed to the analogy with the light velocity constraint in special relativity. This analogy was then emphasized by Bohr [44,45]. In this paper, Bohr used the term “reciprocal uncertainty” for the Heisenberg uncertainty relation. (This reciprocity is related to position and momentum).

*“Heisenberg has rightly compared the significance of this law of reciprocal uncertainty for estimating the self-consistency of quantum mechanics with the significance of the impossibility of transmitting signals with a velocity greater than that of light for testing the self-consistency of the theory of relativity. …Planck’s discovery has brought before us a situation similar to that brought about by the discovery of the finite velocity of light.”*

For the formulation of the complementarity principle, the concrete value of the Planck constant is not important. It is important only that this quantum of action exists, h≠0. In the same way, the concrete value of light’s velocity is not important for formulation of special relativity, i.e., it is only important that it is finite, c<∞. We also stress that constancy of action quantum, its independence of observable (measurement procedure), plays the crucial role in QM, as well as the constancy of the speed of light in special relativity.

What are the other principles of quantum theory? We shall discuss this problem in Section 8.

## 8. Bohr’S Complementarity Principle

In 1949, Bohr [30] presented the essence of complementarity in the following widely citing statement:

*“This crucial point …implies the impossibility of any sharp separation between the behaviour of atomic objects and the interaction with the measuring instruments which serve to define the conditions under which the phenomena appear. In fact, the individuality of the typical quantum effects finds its proper expression in the circumstance that any attempt of subdividing the phenomena will demand a change in the experimental arrangement introducing new possibilities of interaction between objects and measuring instruments which in principle cannot be controlled. Consequently, evidence obtained under different experimental conditions cannot be comprehended within a single picture, but must be regarded as complementary in the sense that only the totality of the phenomena exhausts the possible information about the objects.”*

By analyzing this Bohr’s statement, I propose [1,3,51] to present the Bohr complementarity principle as constituted by the following three interconnected principles:

- **Contextuality:** Irreducible dependence of measurement’s output on the experimental context.
- **Context complementarity:** Existence of complementary experimental contexts.
- **Individuality:** Discreteness of quantum measurements -generation of physical phenomena.

In this formulation, the complementarity principle can be treated as an epistemological principle (see, especially, Reference [51] on coupling to quantum information theory). I would emphasize that this my personal representation of Bohr’s ideas on complementarity (see Section 10 for a further discussion).

Typically, one identifies the Bohr complementarity principle with **Context complementarity**. However, the above citation implies combination of all four “sub-principles.” Besides **Context complementarity**, the principle **Contextuality** also attracts some attention, but **Individuality** is completely ignored, although it plays the crucial role in distinguishing quantum theory from e.g. classical electromagnetism (see Reference [4]). By this principle, quantum measurements generate discrete events corresponding to interaction of individual quantum systems, say photons or electrons, with measuring devices. Such discrete events are clicks of photo-detectors or points on a screen with photo-emulsion in the original diffraction experiments. Bohr called these phenomena. For him, only phenomena can be considered as “elements of reality”. We now cite Bohr:

*“I advocate the application of the word* **phenomenon** *exclusively to refer to the observations obtained under specific circumstances including an account of the whole experimental arrangement. In such terminology, the observational problem is free of any special intricacy since, in actual experiments, all observations are expressed by unambiguous statements referring, for instance, to the recording of the point at which an electron arrives at a photographic plate. Moreover, speaking in such a way is just suited to emphasize that the the appropriate physical interpretation of the symbolic quantum mechanical formalism amounts only to predictions, of determinate or statistical character, pertaining to individual phenomena appearing under conditions defined by classical physical concepts.”*

(Reference [30], v. 2, p. 64)

This, it seems that Zelinger’s principle of information quantization is just an information reformulation of Bohr’s principle of individuality of quantum phenomena.

Besides **Individuality**, in the above citation Bohr also emphasized **Contextuality**, *an account of the whole experimental arrangement.* We remark that, for to Bohr, **Contextuality principle** is a consequence of **Quantum action principle.** The indivisibility of quantum of action implies irreducible dependence of measurement’s output on the experimental context. Logically **Contextuality** should generally imply **Context complementarity**, since the possibility to combine any group of experimental contexts into a single context for join measurement of observables is really surprising. For me, the real surprise is not that some experimental contexts are incompatible, e.g., contexts for measurement of position and momentum in QM, but that, in some theories, e.g., classical physics, mutual compatibility of any pair of contexts is always assumed.

We point out that, in discussions related to violation of the Bell type inequalities, the term “contextuality” is used in the very restricted meaning, as dependence on measurement of a compatible observable [15]. In the present paper, as well as in my previous works, e.g., Reference [73], “contextuality” was used to note dependence on a general experimental context (“whole experimental arrangement”). To speak about contextuality, we need not to consider two observables; we can speak, e.g., about the context of position measurement or the context of measurement of the concrete spin projection. This general contextuality can be called *Bohr contextuality* [74].

## 9. The Fundamental Principles of Quantum Mechanics

The above considerations lead in a new way to the fundamental principles of QM:

1. **Quantum action principle.**
2. **Bohr’s complementarity principle.**

We consider QM as an epistemic theory [49,50,55,61,62], a theory about extraction of knowledge about nature; in the terminology of Hertz and Boltzmann this is an observational theory [56,75,76,77]. Together, these two principles provide the epistemic foundations of quantum theory.

The quantum action principle is the direct consequence of the quantum postulate (the ontic principle about nature as it is), the second quantum principle (complementarity) is based on the first quantum principle. But their interrelation is complicated (see References [1,3,51]).

Of course, these principles do not provide representation of QM as a closed formal system. In spite of a rather common opinion, even Einstein’s relativity based on the principle of relativity and constancy of light’s velocity cannot be treated as such a closed system; see Einstein’s own comment on this issue (citation is taken from Reference [53]):

*“The principle of relativity, or, more exactly, the principle of relativity together with the principle of the constancy of the velocity of light, is not to be conceived as a “complete system,” in fact, not as a system at all, but merely as a heuristic principle which, when considered by itself, contains only statements about rigid bodies, clocks, and light signals. It is only by requiring relations between otherwise seemingly unrelated laws that the theory of relativity provides additional statements.”*

## 10. Ontic and Epistemic Viewpoints on Complementarity

This section may be of some interest for the readers interested in the philosophic foundations of QM; other readers can jump directly to the section concluding remarks.

We recall that an ontic theory is about reality as it is-when nobody is looking to it, an epistemic theory is about knowledge which can be extracted from observations (see Atmanspacher [49] for details). The separation of ontic and epistemic theories is very important for quantum foundations. The majority of quantum paradoxes and mysteries come from mixing the elements of ontic and epistemic theories. According to Bohr, QM is an epistemic theory, it is not about micro-reality as it is, but about measurements performed on micro-systems. Theories with hidden variables are ontic ones. It is important to understand that the ontic representation of reality is not given by the God, but it is also a product of human mind. This viewpoint was especially clear presented in works of Herz and Boltzmann [75,76,77,78]. They consider descriptive and observational theories. These are analogs of ontic and epistemic theories in the sense of Atmanspacher.

By accepting the ontic-epistemic approach to foundations of QM, one understands that ontic and epistemic theories have to be created in pairs, $(T_{\mathrm{ontic}},T_{\mathrm{epistemic}}),$ and that the selection of proper mapping

(3) $$J:T_{\mathrm{ontic}}\to T_{\mathrm{epistemic}}$$

plays the crucial role.

The main problem of Bell’s argument is precisely mixing of ontic and epistemic descriptions and the use of straightforward mapping (3) (even without explicit pointing to it) identifying ontic and epistemic quantities. By using the ontic-epistemic approach and selecting the right corresponding mapping, one can overcome Bell’s, as well as von Neumann’s, no-go statements (see, e.g., References [79,80,81,82] and Appendix B). Typically, mapping (3) has two counterparts, one for states and another for quantities (in the epistemic theory, quantities are identified with observables); denote them $J_{\mathrm{state}}$ and $J_{\mathrm{quantity}}.$ The possibility to construct (theoretically) these maps smooths a dichotomy of ontic and epistemic descriptions (emphasized by Atmanspacher).

In particular, for injective mapping $J_{\mathrm{quantity}},$ the preimages of incompatible observables *A* and *B* (represented in QM by noncommuting operators, [A,B]≠0) are well defined elements *a* and *b* of $T_{\mathrm{ontic}}$ These preimages lose the property of mutual exclusivity. However, they have some special properties leading to probability interference within $T_{\mathrm{ontic}}.$ We call them supplementary (see Reference [83] for details; see Norris [84] for coupling with Derrida’s logic of supplement [85]). The supplement is understood as supplying what is missing and in this way is already inscribed within that to which it is added.

The early Bohr considered the complementarity principle as an epistemological principle, as a principle about extraction of information about features of quantum systems with the aid of measurement devices. In fact, Bohr’s views match very well with modern development of quantum information theory (see Plotnitsky [31,32], Jaeger [86,87]), including the information interpretation of QM (Zeilinger-Brukner [48,88,89]), QBism [90,91], and information reconstruction of quantum theory [57,58,59,60]. The late Bohr’s treatment of the complementarity principle is closer to ontic.

The discussion on ontic and epistemic counterparts of Bohr’s complementarity principle can be concluded with the following remark. The Franco-Romanian philosopher and logician Stephane Lupasco, writing in the 1950s–1960s, developed a non-propositional logic (Logic in Reality, LIR) capable of describing the evolution of onto-epistemic processes at all levels of reality. In his analysis of Bohr, (see Brenner [92]), Lupasco stated that Bohr’s early work indicates that he viewed complementarity as primarily an epistemological principle [46]:

*“The very nature of quantum theory forces us to regard space-time co-ordination and causality, the union of which characterizes classical theories, as complementary but exclusive features of the description, symbolizing the idealization of observation and definition respectively.”*

*“The term complementarity, which is already coming in to use, may perhaps be more suited also to remind us of the fact that it is the combination of features which are united in the classical mode of description but appear separated in quantum theory that ultimately allows us to consider the latter as a natural generalization of the classical physical theories.”*

Later, Bohr seems to have moved toward a more ontological interpretation: phenomena or information were mentioned as being complementary, rather than descriptions.

*“The phenomenon by which in the atomic domain objects exhibit the properties of both particles and waves that in classical, macroscopic physics are mutually exclusive categories.”*

According to Brenner [92], if the fundamental nature of dynamic antagonism is accepted, a real contradictorial relation in quantum phenomena is neither physically nor logically unacceptable, and it can have both epistemological and ontological aspects. It is not physically unacceptable because wave and particle properties are not fully instantiated at the same time, until the measurement of one potentializes the other. It is not logically unacceptable for exactly the same reason. Two answers can be given to the objection that this formulation simply restates the result of experiment: (1) if the particle aspects are actualized, the wave aspects must be present as potential, and vice versa; otherwise, it is difficult to explain how they could reappear; (2) it is not in the LIR view that there is any problem with the observed duality of quantum entities in the first place (also see Reference [93]).

## 11. Concluding Remarks

From my viewpoint (and I hope that the arguments presented in this paper support this viewpoint),

- the EPR [13] was not directed against the Heisenberg uncertainty and Bohr complementarity principles; and
- Bell’s work, see, e.g., References [14,15,16] and further works following Bell’s paradigm, e.g., Reference [17], were straightforwardly directed against these principles.

However, Bell believed [14] that he is in the boat with EPR. And this belief spread throughout the quantum community.

The recent years have been marked by the tremendous success of experimentalists performing Bell type tests [68,69,70]. In the light of this paper (as well as Reference [1]), these tests can be considered as an excellent confirmation of the validity of the Bohr complementarity principle. They also confirmed that the correlations predicted within quantum theory can be preserved at long distances. In this paper, we do not try to provide “deeper explanation” of these correlations than given by the quantum formalism (see a short remark at the very end of Appendix A). We just wanted to point that the attempt of their “explanation” in the Bell framework was suspicious from the very beginning (i.e., without derivation of any inequality), as an attempt to disprove the complementarity principle.

The ontological core of the complementarity principle is the quantum action postulate. Therefore rejection of complementarity is impossible without rejection of the existence of an indivisible quantum of action.

Following Reference [48], we searched for the fundamental principles of QM. These are two, the quantum action and complementarity principles. The first principle is the epistemological representation of the quantum action postulate.

Finally, I conclude that if the foundation of quantum theory are presented as in Section 7, i.e., similarly to the foundations of special relativity, then *the attempts to go beyond the complementarity principle, e.g., with hidden variables of the Bell type, can be compared with the attempts to go beyond special relativity, by rejecting Einstein’s principle on the constancy of the speed of light.*

## Appendix A. Janus of Quantum Nonlocality

The first time Lüders nonlocalty was briefly mentioned in EPR [13] as the absurd alternative to incompleteness of QM endowed with the Copenhagen interpretation.

During the Einstein-Bohr debate non of the debaters considered this alternative seriously. Unfortunately, Einstein mentioned nonlocality at a few other occasions and highlighted it in Reference [10] with the catchy slogan, “spooky action at a distance.” This sort of nonlocality is the straightforward consequence of using the projection postulate in combination with the Copenhagen interpretation: the quantum state is the state of the individual quantum system. We remark that the Copenhagen interpretation has many versions. Plotnitsky even proposed to speak about the interpretations in the spirit of Copenhagen. We characterize such interpretations by emphasizing the individual character of a state. The alternative interpretation is the statistical or ensemble interpretation. Here, the quantum state characterizes the features of an ensemble of identically prepared quantum systems.

**Lüders nonlocalty:** The state update as a retro-action of measurement is mathematically formalized by the Lüders projection postulate. For a compound system $S=(S_{1},S_{2}),$ measurement on $S_{1}$ with the concrete output A=a “instantaneously” modifies the state of $S_{2}.$ Here, the crucial role is played by a meaning of “instantaneously”. In what space? If one follows the individual interpretation of the state, then this instantaneous change happens in physical space. One really can imagine that this instantaneous change is a consequence of spooky action at a distance.

However, as was explained in much detail in Reference [5], if one uses the statistical interpretation of a quantum state, then “instantaneous” is related not to physical space, but to information space. There is nothing special in “instantaneous” change of information. (Here, “information” is treated epistemically).

The same happens in process of probability update in classical probability theory. Here, states of random systems are represented by probability measures. One also might say that such state changes instantaneously. But, nobody describes this situation as nonlocality.

Generally the question of interrelation of classical and quantum probability theories is very complex, its different dimensions were discussed by many authors, in particular, References [31,42,73,87,90,91,94,95,96].

**Bell nonlocality.** This is nonlocality of of some subquantum models invented by Bell and known as models with hidden variables [14,15,16,17]. The existence of such models is not surprising at all, human imagination is powerful and it can generates a variety of mathematical structures that have nothing to do with physics. How does one couple Bell nonlocality with quantum physics? Bell proposed to compare correlations described by subquantum models with quantum correlations. As was pointed out in Reference [5], the Bell project does not take into account the ontic-epistemic structure of scientific theories. Already Hertz [75] and Boltzmann [76,77] (and later Schrödinger [78]) emphasized this difference: descriptive (causal) versus observational theories (also see References [55,56]). Bell tried to identify outputs of the two theories. (This approach was strongly criticized by De Broglie [65]. It seems that he was not aware about the works of Hertz, Boltzmann, and Schrödinger. However, their views in fact coincide).

As was shown in Reference [1], the Bell type inequalities can be considered in the purely quantum framework, as inequalities for correlations described by quantum theory. In this framework, violation versus satisfaction of these inequalities is equivalent to local incompatibility versus compatibility of quantum observables. Hence, Reference [1] demonstrated that these inequalities are statistical tests for the Bohr complementarity principle (in particular, the Heisenberg uncertainty principle).

## Appendix B. Reestablishing Causality within Prequantum Classical Statistical Field Theory-PCSFT

Can one reestablish causality without contradicting to Heisenberg’s uncertainty principle? I think that the answer is “yes”, and the corresponding mathematical model was constructed in a series of my papers; see, e.g., References [79,80,81]. Of course, such reestablishing cannot be done in such a trivial way as in the Bell model with hidden variables, i.e., though simple identification of the values of functions of hidden variables with experimental outcomes.

In References [79,80,81], I developed *prequantum classical statistical field theory* (PCSFT), reproducing quantum probabilities and correlations within theory of classical random fields. PCSFT is a kind of hidden variables model, but the values of classical random variables, functions of classical random fields, are not identified with the outcomes of quantum observables. The PCSFT-counterpart of a quantum observable which is represented by the operator *A* are given by the quadratic form

$$f_{A}\left(\phi \right)=\langle \phi |A|\phi \rangle .$$

The range of values of the quadratci form $f_{A}$ does not coincides with the spectrum of A. In particular, if *A* has the spectrum {−1,+1}, the range of values of $f_{A}$ is not bounded by 1. Correlations of such quadratic forms can violate the Bell-type inequalities. (And this is not surprising).

The basic variables of PCSFT are classical random fields defined on physical space. A random field can be considered as a function of two variables ϕ=ϕ(x;ω): *x* is the spatial variable (with three real coordinates); ω is a random parameter. We remark that random fields can be considered as random vectors valued in the complex Hilbert space $H=L_{2}\left(R^{3}\right)$ of square integrable complex valued functions.

The key point of this theory is that covariance operator *B* of random field ϕ is identified (up to normalization by trace) with the density operator of QM:

(A1) B→ρ=B/TrB.

The covariance operator is an element of the descriptive theory-PCSFT and the density operator is the element of the observational theory (QM). (For a complex valued random field, its covariance operator *B* is a Hermitian positive operator with the finite trace. Thus, *B* has all features of a density operator, besides normalization Trρ=1). Quadratic forms are elements of the descriptive theory-PCSFT and Hermitian operators are elements of the observational theory QM.

We remark that here the trace of field’s covariance operators equals to average of field’s energy:

(A2) $$\mathrm{Tr}B={E∥\phi \left(\omega \right)∥}^{2},$$

where *E* is mathematical expectation, and

$${∥\phi \left(\omega \right)∥}^{2}=\int_{R^{3}}{\left|\phi \left(x;\omega \right)\right|}^{2}\;dx$$

is square of the $L_{2}$-norm of the field (for the concrete value of the random parameter ω).

PCSFT is a causal descriptive theory beyond the observational theory-QM (see Hertz and Boltzmann [75,76,77], as well as References [55,56]). PCSFT can be straightforwardly connected with observations through mapping onto QM. However, if one is looking for causal coupling with observations, then PCSFT has to be endowed with its own observation theory. Such a theory should describe generation of measurement outputs from quadratic forms $\phi \to f_{A}\left(\phi \right).$ The first steps towards such PCSFT-based measurement theory were done in Reference [81]. This measurement theory is based on detectors of the threshold type. It does not violate the Heisenberg uncertainty nor the Bohr complementarity principles. (However, the role of indivisible quantum of action in this theory has not yet been clarified.) In particular, the PCSFT-generated observational model reproduces violation of the CHSH inequality for discrete clicks of detectors (of the threshold type) [80]. This model is non-trivially coupled with the temporal structure of measurements, in particular, their constraining by time-coincidence of detections for Alice’s and Bob’s detectors.
